# Death of Mrs. Jordan W. Lambert

**Published:** 1889-04

**Authors:** 


### DEATH OF MRS. JORDAN W. LAMBERT.

Scarcely had subsided the shock to his large circle of friends, both in and out of the profession, caused by the announcement of the death of Jordan W. Lambert, of St. Louis, before we are called upon to chronicle the death of his young and accomplished wife, Mrs. Lillie Lambert, *nee* Wynne. She died in St. Louis, March 31, ult., and was laid by the side of her husband on the 1st, inst

Mrs. Lambert was a model woman, and combined in her character, rare traits of benevolence and charity, love and sympathy; and in every relation of life, in society, in the home circle, and in the church, was an ornament; her character was most exemplary. As a mother to her young charge of five,—four sons and one daughter—all under fourteen years of age, the beauty of her true womanhood shone most resplendent. Oh, who shall comfort the tender flock so sadly bereaved, but Him who tempereth the winds to the shorn lamb? May He take them into His special keeping, and guard them against those evils which she had so sedulously taught them to avoid.

STILL BORN—As predicted by this JOURNAL the *individual* bill for a State Board of Health presented to the 21st Legislature and to the T. S. M. A. Committee on Legislature—not by any association, but sent to Senator Woodward in a letter over an individual signature with instructions to introduce it—not only failed to pass but failed *to receive any notice whatever* at the hands of the Legislature; it was read and referred to Committee, and, there it was pigeon-holed; never saw day-light again!

We omitted to state in our last issue that a majority of the members of the Committee on Legislation being importuned to adopt it, by those who had cut-out the work ready to their hands, unasked—and help it through, met inAustin and formally declined to touch it,—even with a pair of tongs.

This action though not *loud* was significant; a liberal interpretation of it might be construed into, "when we want your assistance we will ask for it."

Our remarks in last issue were by no means intended for a large class of physicians who advocated the passage of a bill—*not knowing* that the State Association had a committee in the field especially entrusted with the important work of framing and pre senting a bill at their discretion, but were intended for those who *did* know this, and yet were ambitious to figure in the matter,—even unasked. We do not understand that the bill in question emanated from any medical or sanitary organization; we saw the letter accompanying it, and it bore the signature as well as the *ear* marks of a certain *loud* party, "*nameles shere forever more.*"

We, in common with most of the members of the State Association are in favor of a Board of Health, or of some addition to the present so called health system, whereby the important feature of vital statistics can be looked after; but as the matter has been left to a committee we must await their report, at least, before any other action can well be taken.

---

The Committee of Arrangements at the appointed place of meeting of the Texas State Medical Association have usually procured reduced rates on the railroads and at hotels, and has also usually sent out in advance of the meeting, notices to members covering these and other points of interest. Just why this has not been done in the present instance by the committee at San Antonio is hard to conjecture. The Journal, ever desirous to post members on all points of interest in connection with an approaching meeting, wrote twice to San Antonio to members of the Committee asking for the railroad and hotel rates for publication in this issue,—which will reach readers just before the meeting, but for some reason said members failed to furnish the desired information. The Journal is devoted to the interests of the Association and would have been glad either on its own account to give readers this information, or to allow the Secretary to publish it officially; but the Secretary was not even made acquainted with the action of the Committee, if any action has been taken on the subject.

It is the information of the JOURNAL that Dr. P. W. Johns, President West Texas Medical Society, will deliver the address of welcome to the Association on behalf of that Society, and in the name of the profession of that city. This is the only details of the arrangements at this late day imparted to the Secretary and to this JOURNAL.

---

A PUBLIC TRIAL OF SURGICAL PROWESS!—The trial of gynecological skill shortly to come off for the championship of America and, we suppose, "the belt", is a a departure which should not be countenanced by the profession. It savors decidedly of the prize-ring, and suggests the "Marquis of Queensbury rules." We suppose the affair will be duly chronicled in all its details, and may expect such announcements as "first blood for Doctor Ballyrag"—"Doctor Gallypop got in a right-hander, followed by an ugly upper cut." We do not think any credit attaches to the affair, and it is an unfortunate occurrence altogether.

---

DR. DAY'S LETTER will be read with interest. It is singular at least, that American journals are so prone to magnify and proclaim every discovery, and advance in medicine which emanates from Germany—and seldom take notice of anything which originates at home, especially in the South. The question has been asked, "can any good come out of Nazareth?" Northern journals seem to think that nothing good can originate in Texas or Louisiana. "Honor to whom honor is due," should be observed.

---

WE OBSERVE, that Dr. C. M. Rosser, late of Waxahachie, has removed to Dallas, and acquired an interest in the *Texas Courier Record of Medicine*,—becoming joint editor with Dr. Brooks, of that sprightly periodical. The Doctor makes his bow with becoming modesty and we bespeak for him a liberal support in his new field of labor.

